# Y-chromosome evidence suggests a common paternal heritage of Austro-Asiatic populations

**DOI:** 10.1186/1471-2148-7-47

**Published:** 2007-03-28

**Authors:** Vikrant Kumar, Arimanda NS Reddy, Jagedeesh P Babu, Tipirisetti N Rao, Banrida T Langstieh, Kumarasamy Thangaraj, Alla G Reddy, Lalji Singh, Battini M Reddy

**Affiliations:** 1 Molecular Anthropology Group, Biological Anthropology Unit, Indian Statistical Institute, Street No. 8, Hubsiguda, Hyderabad – 500 007, India; 2 Department of Anthropology, North Eastern Hill University, Shillong – 793 014, India; 3 Centre for Cellular and Molecular Biology, Uppal Raod, Hyderabad – 500 007, India

## Abstract

**Background:**

The Austro-Asiatic linguistic family, which is considered to be the oldest of all the families in India, has a substantial presence in Southeast Asia. However, the possibility of any genetic link among the linguistic sub-families of the Indian Austro-Asiatics on the one hand and between the Indian and the Southeast Asian Austro-Asiatics on the other has not been explored till now. Therefore, to trace the origin and historic expansion of Austro-Asiatic groups of India, we analysed Y-chromosome SNP and STR data of the 1222 individuals from 25 Indian populations, covering all the three branches of Austro-Asiatic tribes, viz. Mundari, Khasi-Khmuic and Mon-Khmer, along with the previously published data on 214 relevant populations from Asia and Oceania.

**Results:**

Our results suggest a strong paternal genetic link, not only among the subgroups of Indian Austro-Asiatic populations but also with those of Southeast Asia. However, maternal link based on mtDNA is not evident. The results also indicate that the haplogroup O-M95 had originated in the Indian Austro-Asiatic populations ~65,000 yrs BP (95% C.I. 25,442 – 132,230) and their ancestors carried it further to Southeast Asia via the Northeast Indian corridor. Subsequently, in the process of expansion, the Mon-Khmer populations from Southeast Asia seem to have migrated and colonized Andaman and Nicobar Islands at a much later point of time.

**Conclusion:**

Our findings are consistent with the linguistic evidence, which suggests that the linguistic ancestors of the Austro-Asiatic populations have originated in India and then migrated to Southeast Asia.

## Background

**Figure 1:**
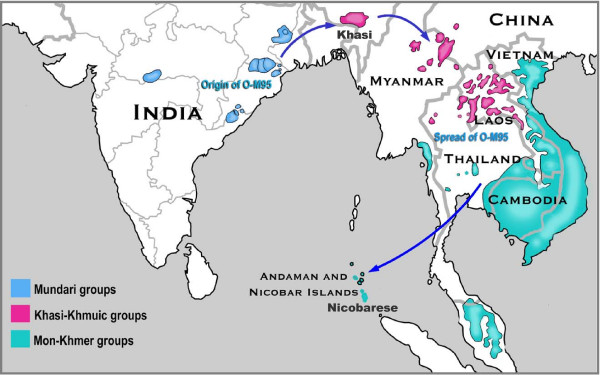
Map showing present-day distribution of Austro-Asiatic groups (modified from van Driem [2]) and the schematic representation of the routes of migration of the different Austro-Asiatic linguistic subgroups of India.

The Indian subcontinent is presently inhabited by four major linguistic groups, viz. Austro-Asiatic, Dravidian, Indo-European and Tibeto-Burman that might have entered at different points of time. Based on the observation that Austro-Asiatic family has the greatest divergence in their nouns [1] and some other linguistic features (for details, refer to discussion), it is considered to be the oldest of the four linguistic families [1,2] and consists of three sub-families [3]: (1) Mundari, spoken by a number of tribes inhabiting Chota-Nagpur plateau in Central and Eastern India, (2) Mon-Khmer, spoken by Nicobarese and Shompen tribes from Andaman and Nicobar islands and (3) Khasi-Khmuic (which linguists earlier considered as part of Mon-Khmer) represented by only the Khasi subtribes from Northeast India (Fig. 1). The Indian Khasi-Khmuic to a certain extent and Mon-Khmer groups have physical features of East Asian populations [4], whereas the Mundari populations have features similar to those of the Dravidian linguistic family. Further, except the Mundari sub-family which is restricted to the Indian subcontinent, the languages of the other two sub-families of Austro-Asiatics are spoken by a large number of populations in Southeast Asia (Fig. 1). However, neither the possibility of any genetic link among the three linguistic branches of Indian Austro-Asiatics, nor that between the Indian and Southeast Asian Austro-Asiatics has been comprehensively explored till now, despite the fact that the Indian subcontinent has been considered to have probably served as an important corridor for migrations to Southeast Asia.

Two routes of migration by which Austro-Asiatic groups possibly entered the Indian subcontinent have been suggested based on the linguistic, archaeological and classical genetic marker [4] and the references therein]; the first being migration from Africa to India via Central Asia, while the second route is from Africa to Northeast Asia and then to the Indian subcontinent. Basu et al. [5] found high frequency of Haplogroup K-M9 among the Mundari populations and inferred that the Austro-Asiatic populations have migrated from Africa to India via central Asia, which is flawed since this haplogroup is ubiquitously found in Asia and has a substantial presence in the whole of East Asia. On the other hand, from the analysis of mtDNA 9bp (9-base-pair) del/ins (deletion/insertion) polymorphisms, Thangaraj et al. [6] and Prasad et al. [7] reported only East Asian-specific mtDNA haplogroups in Nicobarese, while Roychoudury et al. [8] and Metspalu et al. [9] found only Indian-specific mtDNA haplogroups in Mundari populations. The above inferences were, however, based on meager genetic evidence and very few Austro-Asiatic populations (a maximum of 3) were included in those studies. Although Kumar et al. [10] analysed a large number of Austro-Asiatic populations and suggested distinct origins and migration histories of the Mundari, Khasi-Khmuic and Mon-Khmer populations, the analysis was based only on the mtDNA 9bp del/ins polymorphisms and its characterization.

We sampled almost all the Austro-Asiatic populations of India covering the entire geographic and micro-linguistic heterogeneity inherent among them (Table 1 and Fig. S1 [see Additional file 1]). This includes molecular genetic data on the Austro-Asiatic Khasi from Northeast India, which is considered an important corridor for human migrations to Southeast Asia. We present results based on the analysis of Y-chromosome SNP and STR data of Austro-Asiatic tribes along with the previously published data of 214 other relevant populations and try to trace the origin and historic expansion of Austro-Asiatic groups of India. Based on this evidence, we propose that haplogroup O-M95 has originated in the Indian Austro-Asiatics, particularly among the Mundaris, whose ancestors moved further to Southeast Asia along with this haplogroup.

**Table 1:** Geographical distribution and the Linguistic Affiliations along with sample size of the Twenty Five Studied Populations

**Sl. No.**	**Name of the Populations**	**Linguistic Affiliations**	**Area of Sampling at District Level**	**Sample Size**
1	Santhal	Munda, North Munda, Kherwari, (AA)	Jamshedpur from Jharkhand; Purulia from West Bengal	109
2	Bhumij	Munda, North Munda, Kherwari, (AA)	Jamshedpur from Jharkhand; Purulia from West Bengal;	89
3	Mudi	Munda, North Munda, Kherwari, (AA)	Jamshedpur from Jharkhand; Purulia &Midnapore from West Bengal	37
4	Mahali	Munda, North Munda, Kherwari, (AA)	Jamshedpur from Jharkhand; Purulia &Midnapore from West Bengal	25
5	Asur	Munda, North Munda, Kherwari, (AA)	Lohardagga &Gumla from Jharkhand	55
6	Birjia	Munda, North Munda, Kherwari, (AA)	Lohardagga &Gumla from Jharkhand	24
7	Birhor	Munda, North Munda, Kherwari, (AA)	Lohardagga, Gumla &Simdega from Jharkhand; Purulia from West Bengal; Mayurbhanj from Orissa	38
8	Munda	Munda, North Munda, Kherwari, (AA)	Lohardagga, Gumla &Simdega from Jharkhand; Purulia from West Bengal; Mayurbhanj from Orissa	53
9	Ho	Munda, North Munda, Kherwari, (AA)	Mayurbhanj from Orissa	79
10	Korwa	Munda, North Munda, Kherwari, (AA)	Simdega from Jharkhand; Surguja from Chattisgarh	42
11	Korku	Munda, North Munda, Korku, (AA)	Amravati from Maharashtra	59
12	Juang	Munda, South Munda, Kharia-Juang, (AA)	Keonjhar from Orissa	49
13	Kharia	Munda, South Munda, Kharia-Juang, (AA)	Simdega from Jharkhand; Purulia from West Bengal; Mayurbhanj from Orissa	36
14	Savar	Munda, South Munda, Sora-Juray-Gorum, (AA)	Jamshedpur from Jharkhand; Purulia from West Bengal; Mayurbhanj from Orissa	47
15	Lodha	Munda, South Munda, Sora-Juray-Gorum, (AA)	Midnapore from West Bengal	47
16	Oraon	Northern, Kurux, (Dra)	Lohardagga &Gumla from Jharkhand; Surguja from Chattisgarh	91
17	Nagesia	Northern, Kurux, (Dra)	Surguja from Chattisgarh	14
18	Paharia	Northern, Malto, (Dra)	Jamshedpur from Jharkhand; Purulia from West Bengal;	11
19	Pando	Central Zone, Hindi (IE)	Surguja from Chattisgarh	23
20	Kanwar	Central Zone, Rajasthani (IE)	Surguja from Chattisgarh	41
21	Bhuiyan	Eastern Zone, Oriya (IE)	Keonjhar from Orissa	81
22	Bathudi	Eastern Zone, Oriya (IE)	Mayurbhanj from Orissa	36
23	Nicobarese	Mon-Khmer, Nico-Monic, Nicobar, (AA)	Nicobar island from Andaman &Nicobar	11
24	Khasi	Khasi-Khmuic, Khasian, (AA)	West Khasi Hills, East Khasi Hills, Jaintia Hills, Ri-Bhoi from Meghalaya	92
25	Garo	Tibeto-Burman (TB)	South Garo Hill from Meghalaya	33

"AA" Austro-Asiatic; "Dra" Dravidian; "TB" Tibeto-Burman; "IE" Indo-Eurpoean

## Results

### Distribution and frequency of Y-chromosome haplogroups

**Figure 2:**
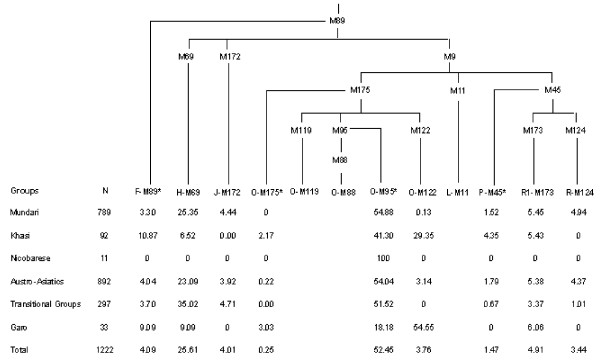
Rooted maximum-parsimony tree of haplogroups defined by binary markers along with their frequency in different groups.

**Figure 3:**
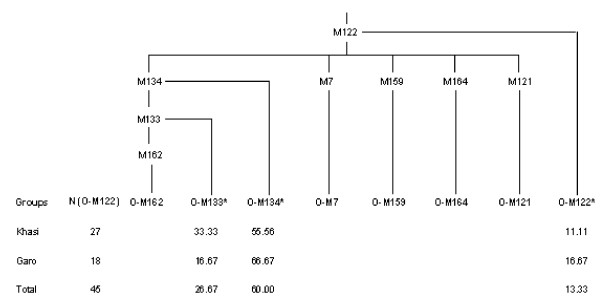
Rooted maximum-parsimony tree of sub-haplogroups of O-M122 along with their frequencies in Khasi and Garo samples.

The population-wise distribution of Y-haplogroup frequency and diversity along with haplotype diversity based on 16 Y-STR is furnished in Table 2. Overall, the haplotype diversity is high (98.87%) and ranges from 95.26% in Pando to 100% in Khasi, Garo, Paharia, Nagesia and Birijia. Out of the 13 potential haplogroups defined by the binary markers typed in the present study (Fig. 2) nine haplogroups were found among these populations. The average frequency of haplogroup O-M95 is highest (52%) followed by H-M69 (26%). Among the three sub-families of Austro-Asiatics, on an average, 55% of Mundari, 41% of Khasi-Khmuic from Northeast India and all the 11 Nicobarese samples belong to O-M95. To know if the unclassified O-M95 samples have sub-linegaes, we also typed downstream M88 binary marker but none showed the presence of O-M88 haplogroups. Except Khasi (29%) and 1 sample of Korku (2%), none of the Indian Austro-Asiatic populations shows the presence of haplogroup O-M122. Further, the Garo tribe shows haplogroup O-M122 as most common (55%) followed by O-M95 (18%). Since Austro-Asiatic Khasi and Tibeto-Burman Garo live in close proximity in Meghalaya and are known to have frequent marital interactions [11,12], we further typed all the samples of haplogroup O-M122 from Garo and Khasi populations to see if O-M122 among the Khasis is not due to admixture with the Garo. We found only 3 out of the 8 haplogroups defined by the binary markers used in this study (Fig. 3) i.e. O-M133*, O-M134* and O-M122*. The frequency of O-M134* was highest in both Khasi (56%) and Garo (67%) followed by O-M133*. Three each of Khasi and Garo out of 27 and 18 O-M122 samples, respectively, remained in the undefined clade O-M122*. Thus the Khasi and Garo show homogeneous distribution of the sub-lineages of O-M122 (χ^2 ^= 1.597; p = 0.45).

**Table 2:** Haplogroup frequencies and Y chromosome diversity

Name of the Population	Sample Size	F-M89*	H-M69	J-M172	O-M175*	O-M122	O-M95	P-M45*	R-M124	R-M173	Haplogroup Diversity +/- SE	Y-STR Haplotype Diversity +/-SE
Santhal	109	0.03	0.39	0.03	0.00	0.00	0.47	0.02	0.02	0.05	0.627 +/- 0.028	0.9913 +/- 0.0039
Bhumij	89	0.02	0.27	0.00	0.00	0.00	0.63	0.00	0.04	0.03	0.534 +/- 0.046	0.9950 +/- 0.0031
Mudi	37	0.03	0.43	0.03	0.00	0.00	0.43	0.03	0.03	0.03	0.640 +/- 0.049	0.9910 +/- 0.0090
Mahali	25	0.04	0.40	0.08	0.00	0.00	0.12	0.00	0.12	0.24	0.777 +/- 0.057	0.9855 +/- 0.0179
Asur	55	0.04	0.22	0.09	0.00	0.00	0.64	0.00	0.00	0.02	0.547 +/- 0.064	0.9970 +/- 0.0045
Birjia	24	0.00	0.00	0.00	0.00	0.00	0.96	0.04	0.00	0.00	0.083 +/- 0.075	1.0000 +/- 0.0120
Birhor	38	0.00	0.24	0.00	0.00	0.00	0.71	0.00	0.00	0.05	0.448 +/- 0.077	0.9640 +/- 0.0189
Munda	53	0.02	0.25	0.00	0.00	0.00	0.45	0.08	0.08	0.13	0.719 +/- 0.044	0.9898 +/- 0.0062
Ho	79	0.00	0.25	0.01	0.00	0.00	0.66	0.04	0.04	0.00	0.506 +/- 0.051	0.9949 +/- 0.0034
Korwa	42	0.00	0.33	0.02	0.00	0.00	0.60	0.00	0.05	0.00	0.545 +/- 0.054	0.9906 +/- 0.0090
Korku^¶^	59	0.07	0.08	0.00	0.00	0.02	0.81	0.02	0.00	0.00	0.331 +/- 0.076	0.9989 +/- 0.0053
Juang	49	0.02	0.00	0.00	0.00	0.00	0.98	0.00	0.00	0.00	0.041 +/- 0.039	0.9745 +/- 0.0114
Kharia	36	0.17	0.33	0.03	0.00	0.00	0.39	0.00	0.00	0.08	0.722 +/- 0.041	0.9982 +/- 0.0077
Savar	47	0.09	0.32	0.13	0.00	0.00	0.15	0.00	0.00	0.32	0.767 +/- 0.030	0.9677 +/- 0.0123
Lodha	47	0.02	0.15	0.32	0.00	0.00	0.09	0.00	0.43	0.00	0.702 +/- 0.039	0.9815 +/- 0.0115
Oraon	91	0.02	0.57	0.00	0.00	0.00	0.32	0.01	0.03	0.04	0.575 +/- 0.039	0.9980 +/- 0.0022
Nagesia	14	0.00	0.36	0.07	0.00	0.00	0.57	0.00	0.00	0.00	0.582 +/- 0.092	1.0000 +/- 0.0302
Paharia	11	0.00	0.64	0.00	0.00	0.00	0.36	0.00	0.00	0.00	0.509 +/- 0.101	1.0000 +/- 0.1265
Pando	23	0.04	0.22	0.09	0.00	0.00	0.65	0.00	0.00	0.00	0.542 +/- 0.101	0.9526 +/- 0.0335
Kanwar	41	0.17	0.29	0.15	0.00	0.00	0.39	0.00	0.00	0.00	0.729 +/- 0.034	0.9892 +/- 0.0102
Bhuiyan	81	0.01	0.11	0.04	0.00	0.00	0.84	0.00	0.00	0.00	0.285 +/- 0.062	0.9959 +/- 0.0031
Bathudi	36	0.00	0.39	0.06	0.00	0.00	0.36	0.03	0.00	0.17	0.706 +/- 0.041	0.9964 +/- 0.0082
Nicobarese^#^	11	0.00	0.00	0.00	0.00	0.00	1.00	0.00	0.00	0.00	0.000 +/- 0.000	0.9643 +/- 0.0772
Khasi	92	0.11	0.07	0.00	0.02	0.29	0.41	0.04	0.00	0.05	0.730 +/- 0.030	1.0000 +/- 0.0031
Garo	33	0.09	0.09	0.00	0.03	0.55	0.18	0.00	0.00	0.06	0.669 +/- 0.077	1.0000 +/- 0.0171

Total	1222	0.04	0.26	0.04	0.00	0.04	0.52	0.01	0.03	0.05	0.533^§^	0.9887^§^

^¶^Unpublished data form Dr. V. R. Rao, Anthropological Survey of India; ^#^SNP data from Thangaraj et al. [40]; ^§^Average

### Analysis of Molecular Variance and Y-STR Networks

The Analysis of Molecular Variance (AMOVA) based on Y-SNPs (Table 3) suggests that Indian and Southeast Asian Austro-Asiatic populations are well differentiated (F_ST _= 0.203). The F_ST _value is larger by 3% between Mundari and Southeast Asian Austro-Asiatics indicating relatively higher level of differentiation between them. However, the F_ST _value turns out to be relatively much smaller (0.045) between Khasi and Southeast Asian Austro-Asiatic populations, suggesting greater genetic affinity between them when compared with the Khasi affinity to Mundari populations (F_ST _= 0.099). Nicobarese were not included in this analysis as all their samples fall in O-M95. AMOVA based on Y-STRs suggests high F_ST _value (0.175) between Mundari and Khasi, but relatively much smaller when compared to that between Khasi and Nicobarese (0.289) or between Mundari and Nicobarese (0.442).

**Table 3:** Analysis of Molecular Variance (AMOVA)

Groups	F_ST_	
	
	Y-SNP	Y-STR
Munda Vs Khasi	0.099	0.175
Munda Vs Nicobarese	-	0.442
Khasi Vs Nicobarese	-	0.289
Munda Vs S.E. AA	0.227	-
Khasi Vs S.E. AA	0.045	-
Indian AA Vs S.E. AA	0.203	

S.E. AA = South East Asian Austro-Asiatic.

Note.- All the p-values are < 0.05.

Since the entire Nicobarese sample falls into single haplogroup, AMOVA based on Y-SNP was not performed. Y-STR data for Austro-Asiatic populations of Southeast Asia was not available.

**Figure 4:**
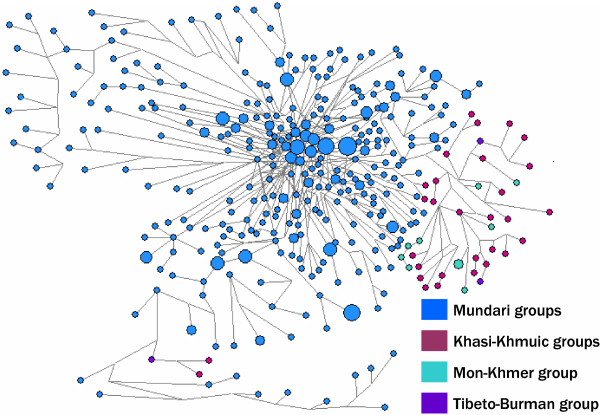
Median-Joining network of Y-STR haplotypes of O-M95 haplogroup. Samples with data on some STRs missing were excluded and the remaining 564 chromosomes were analysed. Circles represent haplotypes with area proportional to their frequency. Microsatellite mutations are represented by black lines.

The Median Joining (M-J) network based on the 16 Y-STRs of the O-M95 samples (Fig. 4) depicts two broad and distinct clades, one representing Mundari groups and the other Khasi and Nicobarese populations. Although the Nicobarese clade is a part of the Khasi clade, it consists of two distinct branches suggesting a separate identity. As expected, due to the considerable degree of admixture, the M-J network shows that the Garo samples form a part of Khasi subclade, not a separate clade. Further, the M-J network constructed separately for the sub-haplogroups of O-M122 suggests that neither in the case of O-M134* (Fig. S2 [see Additional file 1]) nor in the case of O-M133* (results not shown) the Garo and Khasi samples form distinct clades, suggesting a distinct possibility of gene flow between them.

### Time to the most recent common ancestor (TMRCA)

Since O-M95 was found to be the most common haplogroup, we estimated TMRCA for this lineage with the help of BATWING program [13] and using 16 Y-STRs. We chose the demographic model with an exponential growth from an initially constant-sized population with sub-division. Although there are other 2 models, the demographic model chosen for this study is considered to be the most appropriate [14-17] for human populations; this model assumes that population sizes were constant until they became semi-sedentary or sedentary, probably till the advent of agriculture, which was followed by an exponential growth. (for details on computational procedure, see Methods). The median estimate of TMRCA (Table 4) with 10^6 ^MCMC cycles for the whole of Austro-Asiatic groups turns out to be ~68,000 YBP (95% C.I. 25,442 – 132,230). The TMRCA for the Mundari (~66,000) and Khasi (~57,000) is similar, whereas for Nicobarese it is considerably lower (~17,000). Further, the average of TMRCA estimated for individual Mundari and transitional populations turns out to be large (~48,000), ranging from ~70,000 YBP to ~30,000 YBP (results not shown), suggesting that the haplogroup O-M95 might have originated early, possibly in the Paleolithic period.

**Table 4:** Estimates of TMRCA for haplogroup O-M95 according to sub-linguistic families

Groups	TMRCA (Years)	95% Confidence Interval (years)	
		Lower Bound	Upper Bound

Munda	65,730	25,442	132,230
Khasi	57,252	27,644	92,201
Nicobarese	16,578	4,565	51,377
Austro-Asiatic	68,098	25,992	146,833

### Haplogroup isofrequency maps

**Figure 5:**
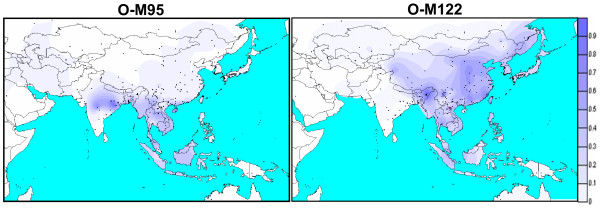
The isofrequency maps portraying spatial distribution of Haplogroups in Asia and Oceania for O-M95 and O-M122 (data are from [14-16, 18-30]). For O-M95, Nicobarese samples were excluded. The dots indicate the populations and the regions from where it was sampled.

Isofrequency maps were generated for all the haplogroups but only the relevant two maps pertaining to O-M95 and O-M122 are presented in Figure 5. The data of our study along with the comparative data on 214 other relevant populations suggest that the haplogroup O-M95 is ubiquitously found in Southeast Asia, while in India it is restricted to the regions where Austro-Asiatic populations are found. This strongly suggests that Austro-Asiatic populations of India are not only linguistically linked to Southeast Asian populations but also genetically associated. The present day distribution of Austro-Asiatic linguistic groups and the distribution of haplogroup O-M95 appear to be highly correlated (Table 5 and Fig. 5). For example, its average frequency is only 3.4% and 0.1% (Table 5), respectively, in northeast and Central Asia where no Austro-Asiatic population is found, whereas it is much higher in Southeast Asian Austro-Asiatics (38%) as well as in the neighboring non-Austro-Asiatics (14.7%). Further, this frequency is significantly much higher in Austro-Asiatics than in non-Austro-Asiatics (χ^2 ^= 22.77; P < 0.001). There is also a decreasing gradient of O-M95 frequency as we move from India to Southeast Asia, although this trend is less apparent in the map because 7 of 45 groups from Southeast Asia show O-M95 frequency in the range of 50% to 75%. However, for six of those 7 populations the sample sizes are less than 20, some being very small. In any case, the average frequency of O-M95 in Indian Austro-Asiatic populations is much higher (54%) when compared (Table 5) to those of Southeast Asia, whether Austro-Asiatic (38%; χ^2^= 68.89; p < 0.001) or non-Austro-Asiatic (14.7%; χ^2 ^= 330.68; p < 0.001). On the other hand, haplogroup O-M122 distribution in India is confined only to the Northeast India, as depicted in the map by a sharp boundary (Fig. 5), whereas it is equally prevalent in Northeast and Southeast Asia (Table 5). The spread of haplogroup H-M69 (Fig. S3 [see Additional file 1]) appears to be confined to the boundaries of the Indian subcontinent and, therefore, very strongly suggests its origin in the Indian subcontinent.

**Table 5:** Distribution of Haplogroups found commonly in Central and East Asia

	No. of Populations^a^		Average in %age (Range) of different Haplogroups		
Region	O-M95/122	N-LLY22g	O-M95	O-M122	N-LLY22g

Central Asia	21	35	0.1 (0 – 1.6)	2.7 (0 – 12.1)	0.9 (0 – 9.5)
Northeast Asia	83	25	3.4 (0 – 50)	34.1 (0 – 85.1)	9.2 (0 – 42.9)
Southeast Asia (Non-AA populations)	37	5	14.7 (0 – 75)	30.5 (0 – 100)	0.5 (0 – 2.4)
Southeast Asia (AA Populations)	8	5	38.0 (3 – 68)	34.3 (0 – 70.2)	0

^a^The Haplogroup frequency data are collected from references cited in the legend of Figure 5; AA = Austro-Asiatic

## Discussion

### A common genetic heritage of the Austro-Asiatic groups

Mundari populations show O-M95 as the most common haplogroup and only three of the 22 populations – Lodha, Savara and Mahali- show departure from this general trend (Table 2), which appears to be because of their disputed origin [31]. This haplogroup is also found in a relatively high frequency in the Khasi and Nicobarese. It is therefore not surprising that in a recent study [32] all the 12 samples of Shompen from Nicobar islands, like their own linguistic neighbors from the region, the Nicobarese, showed O-M95. This may underscore that the Mundari, Khasi-Khmuic and Mon-Khmer groups of India are not only linguistically related but also genetically linked, probably with a single but relatively broad paternal genetic source. This haplogroup has been reported to be absent or present in low frequency in other linguistic groups of India [20,25-29], suggesting a distinct genetic identity of the Indian Austro-Asiatic populations. On the other hand, while the Austro-Asiatic populations of Southeast Asia show high frequency of O-M95 (average 38%) their neighboring populations also show considerable frequency (14.7%). However, this haplogroup has negligible presence in North and Central Asia (Fig. 5 and Table 5). Thus the predominance of this haplogroup both in Austro-Asiatic populations of India and Southeast Asia and its absence/negligible presence in other Asian populations suggests a common genetic heritage of the people of this linguistic family.

The virtual absence of O-M95 in the Tibeto-Burman populations of India [20,28,29] suggests that the migrations of these populations into India were not accompanied by the O-M95 haplogroup. Therefore, the presence of this haplogroup in the Garo tribe of Meghalaya is due to high degree of gene flow from the neighboring Khasi, which has been facilitated by the matrilocal system of marriage among these two tribes [11,12]. Similarly, the presence of haplogroup O-M122 in the Austro-Asiatic Khasi with relatively high frequency (29%) could be suspected to be due to gene flow from the neighboring Garo, which is substantiated by a similar frequency and composition of subclades of O-M122 between them (χ^2 ^= 1.597; p = 0.45). Concurrently, no separate Y-STR lineages could be identified in the M-J network within the subclades (Fig. S2 [see Additional file 1]). The comparative data suggests that Southeast Asian Austro-Asiatics near the Northeast border of India [33] have either O-M133* or O-M134* subclades (63%), whereas majority of the Austro-Asiatic populations from geographically distant Southeast China and Cambodia [24,33] have O-M159 subclade (65%), suggesting that the Austro-Asiatic populations of different regions have different subclades of O-M122, which are characteristic of the neighboring non-Austro-Asiatic groups, possibly due to extensive admixture. Therefore, the presence of O-M133* and/or O-M134* subclades in the Austro-Asiatic Khasi and other Tibeto-Burman populations [28] including Garo from Northeast India may imply that the O-M122 in Khasi probably had its source in the neighboring Tibeto-Burman groups, particularly from the Garo. Although the foregoing analysis suggests that the Austro-Asiatic populations of India share common genetic ties, a comparative analysis among the sub-families suggests that these populations have separated quite early and are now well differentiated as indicated by the results of AMOVA (Table 3), the M-J network (Fig. 4) and the TMRCA (Table 4).

### Origin of haplogroup O-M95 and expansion of Austro-Asiatic populations

Given the overwhelmingly high frequency of O-M95 in the Austro-Asiatic populations it is most likely that this haplogroup has originated among them. However, the question is whether it has its origin in India or Southeast Asia? The most likely region of origin of a haplogroup can be identified on the basis of two characteristics – the highest frequency and the highest diversity [34]. The maximum frequency of O-M95 among the 8 Southeast Asian Austro-Asiatic populations is only 35% after excluding 3 populations with small sample size. On the other hand, the sample size for Mundari populations is generally large (~35–109, except for two) and the frequency of the haplogroup ranges from 39%–98% with an average of 63% (excluding the three populations with disputed origin), which is significantly higher compared to that of the Southeast Asian Austro-Asiatics (χ^2 ^= 108.60; p < 0.001). Furthermore, the haplotype diversity among the Mundari populations is as high as 99%. Given this and the fact that this haplogroup is nearly absent in other parts of India as well as in Western and Central Asia, one may safely conclude that O-M95 has originated in Mundari populations roughly around 65,000 YBP (95% C.I. 25,442 – 132,230), as suggested by TMRCA. Therefore, the ancestors of present day Mundari populations must have come to India prior to the origin of haplogroup O-M95, probably in the Pleistocene era. This is consistent with the archeological evidence, which suggest human habitation in mainland India during early Paleolithic times [35-37].

Kayser et al. [16], however, suggested Southeast Asian origin of haplogroup O-M95, implying migration of Austro-Asiatic populations from Southeast Asia to India. Based on the presence of East Asian mtDNA haplogroups, Kumar et al. [10] and Thangaraj et al. [6] suggested that the non-Mundari Austro-Asiatic groups of India (Nicobarese and Khasi) have migrated from Southeast Asia. However, no such maternal genetic link between the Mundari tribes [8-10], and those of Southeast Asia was found. Further, our analysis of 1147 samples representing most of the Mundari tribes, including the transitional groups, shows total absence of East Asian mtDNA haplogroups [Kumar V, Reddy BM and Langstieh BT, unpublished results), suggesting different maternal genetic histories of the three sub-families of Austro-Asiatics of India as compared to the common paternal genetic history outlined earlier. How do we account for this discrepancy? Since all the sub-families of Austro-Asiatics have a common paternal genetic link in haplogroup O-M95, which is absent in the case of mtDNA, a predominantly male driven migration of Austro-Asiatic populations appears to be a strong possibility. However, and most importantly, haplogroup O-M122, which is considered to be the signature haplogroup of Southeast Asian populations, is absent among the Mundari populations, whereas any inference on the migration of populations from Southeast Asia is principally based on the presence of haplogroup O-M122 [18,20,33]. The age estimated for haplogroup O-M122 is between 15,000–60,000 YBP [18] whereas it is ~8,000 YBP for O-M95 in Southeast Asia [16]. Therefore, if indeed Indian Austro-Asiatic populations would have migrated from Southeast Asia, then they should have shown the presence of haplogroup O-M122, and/or the TMRCA estimated for O-M95 among the Mundaris of India should have been much lower (Table 4) than what has been obtained (~65,000 YBP), although given the large Confidence Interval. (25,442 – 132,230) this estimate needs to viewed with caution. Nevertheless, the lower bound of our estimate for the Mundari is still higher and non-overlapping with the upper limit obtained by Kayser et al. [16]. Since Kayser et al. [16] have used relatively higher mutation rates and only 7 of the 16 loci, we reanalyzed our data based on those 7 loci and the mutation rate used by Kayser et al. [16] and observed a similar TMRCA (~65,000 YBP) suggesting that the TMRCA of the present study may not be an artifact of large number of loci and low mutation rate. Furthermore, the Mundari populations are considered to be traditionally hunters and food-gatherers and at present they inhabit the areas unfit for cultivation, which may reflect their traditional mode of subsistence. Therefore, migration of Mundari populations during demic expansion of the agriculturalists in the Neolithic era appears improbable as has been suggested for Nicobarese [6]. Based on these evidences, we suggest that the ancestors of present day Mundari populations have migrated to Southeast Asia instead of coming from Southeast Asia. This scenario is also consistent with the inference that Mundari language is grammatically and phonologically the most conservative branch of the Austro-Asiatic family [2,38] and more similar to proto-Austroasiatics than the other branches of this family suggesting that linguistic ancestors of the Mundari populations have originated in India [39]. The foregoing analysis therefore suggests in-situ origin of O-M95 haplogroup, most probably in the ancestors of present day Mundari populations, who might have carried it further to Southeast Asia.

The results of AMOVA (Table 3), M-J Network (Fig. 4) and TMRCA of haplogroup O-M95 (Table 4) suggest an early separation of Mundari and other Austro-Asiatic populations. Due to this early separation, we expected that at least in one of these groups sublineage of O-M95 might have originated. However, none of the groups showed the sublineage O-M88 (Fig. 2). Till now this lineage has been reported from the region of Cambodia and Laos only in 1 sample [40] suggesting probably that this lineage is present with a very low frequency and is probably originated and confined to that region. Therefore, if the sublineage(s) exists, it is probably identified by some other binary marker(s) which is yet to be known. Since the Khasi shows relatively high frequency of O-M122 (29%) and given that populations of Khasi-Khmuic sub-family are concentrated in the regions North of Burma and Thailand (Fig. 1), one may suspect that Khasi-Khmuic populations have migrated from Southeast Asia to India. However, the presence of O-M122 in the Khasi is observed to be due to gene flow from the neighboring Garo, suggesting that this population was initially devoid of this haplogroup. Moreover, Indian mtDNA haplogroups constitute 30% of the mtDNA motifs of the Khasi subtribes, (Reddy BM et al. unpublished manuscript), which are practically absent in their Tibeto-Burman neighbors [8,41]. Therefore, the presence of East Asian mtDNA among the Khasi could be due to gene flow from the neighboring Garo and the other Tibeto-Burman populations which have virtually only East Asian mtDNA haplogroups. This may reinforce the suggestion that Mundari and Khasi-Khmuic populations have separated long back and the latter have probably gone to Southeast Asia, via the northeast Indian corridor, as reflected in their geographic distribution (Fig. 1).

The Nicobarese is also quite distinct from both the Mundari and Khasi-Khmuic tribes as revealed by the AMOVA (Table 3) and M-J network (Fig. 4) based on Y-STRs. This tribe has only East Asian female lineages [6,7] and only O-M95 as male lineages (Fig. 2 and Table 2). We also performed AMOVA based on the same set of 16 Y-STR for Shompen tribe [32], which is also a Mon-Khmer group. The results suggest that Shompen like the Nicobarese are also quite distinct from the Mundari (F_ST _= 0.402) and the Khasi (F_ST _= 0.476). The TMRCA of Nicobarese (~17,000 YBP) and the Shompen (~19,000 YBP), and the distribution of Mon-Khmer populations (Fig. 1), which is confined to lower part of Burma and Thailand, Vietnam and Cambodia suggest that they have migrated from Southeast Asia to India during demic expansion of the agriculturalists in the Neolithic era [42]. The complete absence of O-M122 among them appears to be due to the profound impact of founder effect and subsequent genetic drift, although the ascertainment bias due to small sample size cannot be ruled out.

### Two possible routes of entry of Austro-Asiatics into the Indian Subcontinent

Kumar and Reddy [4] suggested the possibility of ancestors of Indian Austro-Asiatic tribes having migrated from Africa to India via either Northeast Asia through the Northeast Indian corridor or via Central Asia through its Western Indian corridor. The sister-clade of haplogroup O-M175, i.e. haplogroup N-LLY22g, is confined only to Northeast Asia including Russia and Siberia (Table 5) and is absent or found in negligibly low frequency in Central, South and Southeast Asia. Similarly, the haplogroup O-M175 and its subclades are either absent or found in low frequency in South (except among Austro-Asiatics) and central Asia, while it is present in whole of East Asia. Two alternative scenarios can be envisaged: 1) it appears that the modern man had probably migrated from Africa to Northeast Asia via Central Asia, where the haplogroups N-LLY22g and O-M175 might have originated [43,44]. Subsequently, populations carrying haplogroup O-M175 might have migrated to India, where haplogroup O-M95 could have originated and later on spread to Southeast Asia. However, since 100% of the Mundari populations and 30% of the Khasi samples show Indian-specific mtDNA whereas all the Southeast Asian Austro-Asiatic populations have East Asian-specific mtDNA only [45], this migration could have been primarily male driven. 2) In view of this, the possibility of ancestors of Austro-Asiatics migrating from central Asia to India through the western Indian corridor cannot be discounted, as it can account for the presence of contrasting patterns of mtDNA in the Indian and Southeast Asian Austro-Asiatics. Although neither haplogroup O-M175 nor N-LLY22g has been reported from central Asia, many studies have observed reasonably high frequency of haplogroup K-M9* [15,24,26] and it is possible that these samples may fall in the haplogroup defined by the binary marker M214, which connects haplogroups 'O' and 'N' [46]. However, this marker has not been typed in the Central Asian populations. A section of the population might have migrated towards Northeast Asia where haplogroup 'N' originated and another wave towards South Asia and entered India through its western corridor wherein haplogroup O-M175 originated. Haplogroup O-M95 might have evolved subsequently as a predominant male lineage along with the Indian-specific female lineages. Subsequently, a primarily male-driven and rapid migration of these people to Southeast Asia via Northeast India might have resulted in the total absence of Indian-specific mtDNA haplogroups but presence of 100% East Asian motifs in the Southeast Asian Austro-Asiatics. The age estimation of fossils of anatomically modern man excavated from East Asia is not older than 40,000 YBP [18,47,48], which may imply that the earliest possible migration of Austro-Asiatic populations to Southeast Asia is about 40,000 YBP or later. Therefore, the Mundari populations appear to be one of the earliest source of populations from which the Khasi-Khmuic and Mon-Khmer populations have separated quite early and migrated to and settled in Southeast Asia, while another wave of migration, much later, by the Mon-Khmer people from Southeast Asia through Thailand and coastal southern Burma to Andaman and Nicobar Islands can be inferred from the current spread of Mon-Khmer populations (Fig. 1).

## Conclusion

To sum up we conclude that, because of its very high frequency and diversity, haplogroup O-M95 had an in-situ origin among the Indian Austro-Asiatics, particularly among the Mundaris, not in Southeast Asia as envisaged earlier. Given the large estimate of TMRCA, our study suggests that the Mundari populations are one of the earliest settlers in the Indian Subcontinent. It is most likely that these populations have come from Central Asia through the Western Indian corridor and subsequently colonized Southeast Asia, although more data on Y-chromosome and mtDNA are needed from other relevant populations to draw firmer conclusions.

## Methods

### Sampling

Intravenous blood samples were collected from a total of 1222 healthy unrelated individuals belonging to 25 tribal populations out of which 17 are Austro-Asiatic groups and 7 are transitional groups, which are considered to have certain geographical and historical affinities to the former, and also presumed to be formerly speaking Austro-Asiatic languages. We collected blood samples from different Mundari, Khasi-Khmuic and Mon-Khmer speaking tribal groups in such a way that the entire gamut of genetic heterogeneity of the Austro-Asiatic tribal populations in India is comprehensively reflected; we collected samples from different dialectical categories of the Austro-Asiatic tribal populations and also from different geographic units of the same tribe as some of them are distributed in a wide territory. We have also included the Tibeto-Burman speaking Garo tribe of Meghalaya since it inhabits the contiguous and/or overlapping geographic locations of the Khasi with which it is known to have marital interaction. The area of sampling within each state is shown in Figure S1 [see Additional file 1] while the names of populations along with the sample size and district-wise details of sampling are furnished in Table 1. Informed consent was obtained from all the donors before collecting their blood.

### Genetic Analyses

DNA was extracted from these samples using the protocol of Sambrook [49]. The following 20 Y-Single Nucleotide Polymorphisms (SNPs) which are known to detect variations in Asia were screened using direct sequencing (Fig. 3 and 4): M89, M69, M172, M9, M11, M175, M95, M88, M122, M119, M45, M173, M124, M134, M159, M164, M7, M121, M133 and M162. The details of these markers are described elsewhere [44]. Many of the samples were typed with all the binary markers for internal check on the reliability of the typing and also to detect recurrent mutations. We used the nomenclature as suggested and followed by Y-chromosome consortium [50].

We have also typed the following 20 Y-Short Tandem Repeat (STRs) loci: DYS19, DYS385a, DYS385b, DYS388, DYS389I, DYS389b, DYS390, DYS391, DYS392, DYS393, DYS426, DYS437, DYS438, DYS439, DYS447, DYS448, DYS460, H4, YCAIIa and YCAIIb. Y-STRs were amplified by multiplex PCR [51] and were analysed on ABI 3730 sequencer. The GENOTYPER software was used to analyze the fragment size. The fragment sizes of the alleles were converted into repeat units as suggested by Butler et al. [51].

Although Y-chromosome is highly variable, because of the low rate of parallel and back mutation of the binary markers on the non-recombinant part, they are particularly useful for reconstructing and identifying stable paternal lineages that can be traced back in time over thousand of years [18,50]. Further, the smaller effective population size makes Y-chromosome probably the best genetic tool to study early human migrations as bottleneck events that are often associated with such migrations becoming more pronounced [18,50]. Despite natural selection being a potentially important force which may affect the entire Y-chromosome and produce an increase in frequency of a lineage more rapidly than would be expected by drift, the empirical evidence so far is not conclusive [46].

### Statistical Analyses

Since the DYS389II allele length also contains DYS389I, for all statistical analyses a simple subtraction of DYS389I allele length from that of DYS389II was done to avoid the double-counting variation at DYS389I. The subtracted DYS389II allele is named as DYS389b. Since DYS385a and DYS385b, and YCAIIa and YCAIIb alleles could not be assigned to their respective loci, these loci were omitted and further analyses were done based on the remaining16 Y-STRs. The Y-SNP and modified Y-STR data were then analyzed for haplogroup and haplotype diversity, respectively, along with their associated SE by means of the software package ARLEQUIN 3.01 [52]. Analysis of Molecular Variance (AMOVA) was also carried out for both Y-SNP and STR data using ARLEQUIN 3.01 software. Y-STR haplotypes were grouped according to haplogroups and Median Joining (M-J) network were constructed by use of the program NETWORK 3.0 [53]. A weighting scheme was followed on the basis of the molecular variance of each microsatellite in all the chromosomes, with the weight inversely proportional to the variance. The weights assigned were from 2 to 8.

TMRCA of a haplogroup provides an important limitation to its spread implying that this haplogroup must have spread after this time and the population carrying this lineage must have arrived in this region prior to the origin of this haplogroup. Therefore we estimated the TMRCA using Bayesian Analysis of Tree With Internal Node Generation (BATWING) as implemented in Wilson et al. [13]. This program uses a Markov chain Monte Carlo (MCMC) procedure to generate phylogenetic trees and associated parameter values consistent with input data (a set of Y haplotypes), besides genetic and demographic models. The genetic model assumes single-step mutations of the STRs and the demographic model chosen was an exponential growth from an initially constant-sized population with sub-division. Based on the Zhivotovsky et al. [54] evolutionary mutation rate we applied a gamma distribution of (1.47, 2130) as mutation rate for all the 16 Y-STRs. For alpha, beta and N priors the gamma distribution of (2, 400), (2, 1) and (1, 0.001), respectively, was used [15,17]. We have used the generation time of 25 years. In all runs, 13,000 samples of the output were taken and the first 3,000 runs were discarded as burn-in. Thus, all the results are based on 10,000 samples. The number of MCMC iterations between each sample varied between runs from 10^2 ^to 10^5^, so the overall runs ranged from 10^6 ^to 10^9 ^MCMC cycles. Our initial analyses were performed with 10^6 ^MCMC cycles using all the O-M95 Y-chromosomes of all the populations, but the value of median Nposterior (the effective population size before the population began to expand) was ~13,000 which was quite large given that global value is ~5000 [15,17]. We increased the MCMC cycle and found that the Nposterior value and expansion time decrease and increase, respectively, with increase in MCMC cycle and do not stabilize even at 10^9 ^cycles. Therefore, we chose 3–5 Y-chromosomes of haplogroup O-M95 from each population at random to produce a sample subset and observed a convergence of values of Nposterior and expansion time at 10^6 ^and 10^7 ^MCMC cycles. These parameters suggest a value of ~1000 for the effective population size for this region which is consistent with other studies on East Asia [17,18].

Haplogroup frequency data on 214 populations (sources given in the legend to Fig. 7) from whole of Asia, including the Indian subcontinent, Oceania and Australia gathered from the published sources were used along with our data to generate isofrequency maps using the ArcView program of the GIS software. The data points used here are shown as black dots. As the Nicobarese is the only population sampled from that region, it had an overwhelming influence on the contour, hence excluded from the haplogroup isofrequency map calculation.

## Authors' contributions

BMR conceived the project; BMR and VK designed the study and wrote the manuscript. BTL and VK collected the samples; AGR, ANS, BTL, JPB, TNR and VK performed the experiments; VK performed the statistical analysis; BMR, LS and KT contributed reagents and laboratory equipments; all authors read, revised and approved the final manuscript.

## Supplementary Material

Additional file 1 Additional figures. It contains 3 figures; Fig S1) Map of India showing the area of sampling; S2) M-J Network of Y-STR haplotypes of O-M134* haplogroup; S3) Iso-frequency map of O-M69 in Asia. Click here for file

## References

[B1] Gadgil M, Joshi N, Manoharan S, Patil S, Prasad UVS, Balasubramanian D, Rao NA (1998). Peopling of India. The Human Heritage.

[B2] van Driem G (2001). Languages of the Himalayas: An ethnolinguistic handbook of the Greater Himalayan region with a brief introduction to the symbiotic theory of language.

[B3] Diffloth G, Sagart L, Blench R, Sanchez-Mazas A (2005). The contribution of linguistic palaeontology to the homeland of Austro-Asiatic. The Peopling of East Asia: Putting Together Archaeology, Linguistics and Genetics.

[B4] Kumar V, Reddy BM (2003). Status of Austro-Asiatic groups in the peopling of India: An exploratory study based on the available prehistoric, Linguistic and Biological evidences. J Biosci.

[B5] Basu A, Mukherjee N, Roy S, Sengupta S, Banerjee S, Chakroborty M, Dey B, Roy M, Roy B, Bhattacharyya NP, Roychoudhury S, Majumder PP (2003). Ethnic India: A genomic view, with special reference to peopling and structure. Genome Res.

[B6] Thangaraj K, Sridhar V, Kivisild T, Reddy AG, Chaubey G, Singh VK, Kaur S, Agarawal P, Rai A, Gupta J, Mallick CB, Kumar N, Velavan TP, Suganthan R, Udaykumar D, Kumar R, Mishra R, Khan A, Annapurna C, Singh L (2005). Different population histories of the Mundari- and Mon-Khmer-speaking Austro-Asiatic tribes inferred from the mtDNA 9-bp deletion/insertion polymorphism in Indian populations. Hum Genet.

[B7] Prasad BV, Ricker CE, Watkins WS, Dixon ME, Rao BB, Naidu JM, Jorde LB, Bamshad M (2001). Mitochondrial DNA variation in Nicobarese Islanders. Hum Biol.

[B8] Roychoudhury S, Roy S, Basu A, Banerjee R, Vishwanathan H, Usha Rani MV, Sil SK, Mitra M, Majumder PP (2001). Genomic structures and population histories of linguistically distinct tribal groups of India. Hum Genet.

[B9] Metspalu M, Kivisild T, Metspalu E, Parik J, Hudjashov G, Kaldma K, Serk P, Karmin M, Behar DM, Gilbert MT, Endicott P, Mastana S, Papiha SS, Skorecki K, Torroni A, Villems R (2004). Most of the extant mtDNA boundaries in south and southwest Asia were likely shaped during the initial settlement of Eurasia by anatomically modern humans. BMC Genet.

[B10] Kumar V, Langsiteh BT, Biswas S, Babu JP, Rao TN, Thangaraj K, Reddy AG, Singh L, Reddy BM (2006). Asian and Non-Asian Origins of Mon-Khmer and Mundari Speaking Austro-Asiatic Populations of India. Am J Hum Biol.

[B11] Langstieh BT, Reddy BM (1999). The origin and ethnic position of the Lyngngam among the tribes of Meghalaya: An exploratory study. J of the Indian Anthro Soc.

[B12] Langstieh BT, Reddy BM (2004). Ethno-historic and linguistic background of the Lyngngam and its demographic structure. The NEHU Journal.

[B13] Wilson IJ, Weale ME, Balding DJ (2003). Inferences from DNA data: population histories, evolutionary processes and forensic match probabilities. J of the Royal Statistical Society: Series A (Statistics in Society).

[B14] Qamar R, Ayub Q, Mohyuddin A, Helgason A, Mazhar K, Mansoor A, Zerjal T, Tyler-Smith C, Mehdi Q (2002). Y-chromosomal DNA variation in Pakistan. Am J Hum Genet.

[B15] Zerjal T, Wells RS, Yuldasheva N, Ruzibakiev R, Tyler-Smith C (2002). A genetic landscape reshaped by recent events: Y-chromosomal insights into central Asia. Am J Hum Genet.

[B16] Kayser M, Brauer S, Weiss G, Schiefenhovel W, Underhill P, Shen P, Oefner P, Tommaseo-Ponzetta M, Stoneking M (2003). Reduced Y-chromosome, but not mitochondrial DNA, diversity in human populations from West New Guinea. Am J Hum Genet.

[B17] Xue Y, Zerjal T, Bao W, Zhu S, Shu Q, Xu J, Du R, Fu S, Li P, Hurles ME, Yang H, Tyler-Smith C (2006). Male demography in East Asia: a north-South contrast in human population expansion times. Genetics.

[B18] Su B, Xiao J, Underhill P, Deka R, Zhang W, Akey J, Huang W, Shen D, Lu D, Luo J, Chu J, Tan J, Shen P, Davis R, Cavalli-Sforza L, Chakraborty R, Xiong M, Du R, Oefner P, Chen Z, Jin L (1999). Y-Chromosome evidence for a northward migration of modern humans into Eastern Asia during the last Ice Age. Am J Hum Genet.

[B19] Qian Y, Qian B, Su B, Yu J, Ke Y, Chu Z, Shi L, Lu D, Chu J, Jin L (2000). Multiple origins of Tibetan Y chromosomes. Hum Genet.

[B20] Su B, Xiao C, Deka R, Seielstad MT, Kangwanpong D, Xiao J, Lu D, Underhill P, Cavalli-Sforza L, Chakraborty R, Jin L (2000). Y chromosome haplotypes reveal prehistorical migrations to the Himalayas. Hum Genet.

[B21] Su B, Jin L, Underhill P, Martinson J, Saha N, McGarvey ST, Shriver MD, Chu J, Oefner P, Chakraborty R, Deka R (2000). Polynesian origins: insights from the Y chromosome. Proc Natl Acad Sci USA.

[B22] Capelli C, Wilson JF, Richards M, Stumpf MP, Gratrix F, Oppenheimer S, Underhill P, Pascali VL, Ko TM, Goldstein DB (2001). A predominantly indigenous paternal heritage for the Austronesian-speaking peoples of insular Southeast Asia and Oceania. Am J Hum Genet.

[B23] Hammer MF, Karafet TM, Redd AJ, Jarjanazi H, Santachiara-Benerecetti S, Soodyall H, Zegura SL (2001). Hierarchical patterns of global human Y-chromosome diversity. Mol Biol Evol.

[B24] Karafet T, Xu L, Du R, Wang W, Feng S, Wells RS, Redd AJ, Zegura SL, Hammer MF (2001). Paternal population history of East Asia: sources, patterns, and microevolutionary processes. Am J Hum Genet.

[B25] Ramana GV, Su B, Jin L, Singh L, Wang N, Underhill P, Chakraborty R (2001). Y-chromosome SNP haplotypes suggest evidence of gene flow among caste, tribe, and the migrant Siddi populations of Andhra Pradesh, South India. Eur J Hum Genet.

[B26] Wells RS, Yuldasheva N, Ruzibakiev R, Underhill PA, Evseeva I, Blue-Smith J, Jin L, Su B, Pitchappan R, Shanmugalakshmi S, Balakrishnan K, Read M, Pearson NM, Zerjal T, Webster MT, Zholoshvili I, Jamarjashvili E, Gambarov S, Nikbin B, Dostiev A, Aknazarov O, Zalloua P, Tsoy I, Kitaev M, Mirrakhimov M, Chariev A, Bodmer WF (2001). The Eurasian heartland: a continental perspective on Y-chromosome diversity. Proc Natl Acad Sci USA.

[B27] Kivisild T, Rootsi S, Metspalu M, Mastana S, Kaldma K, Parik J, Metspalu E, Adojaan M, Tolk HV, Stepanov V, Golge M, Usanga E, Papiha SS, Cinnioglu C, King R, Cavalli-Sforza L, Underhill PA, Villems R (2003). The genetic heritage of the earliest settlers persists both in Indian tribal and caste populations. Am J Hum Genet.

[B28] Cordaux R, Weiss G, Saha N, Stoneking M (2004). The northeast Indian passageway: a barrier or corridor for human migrations?. Mol Biol Evol.

[B29] Cordaux R, Aunger R, Bentley G, Nasidze I, Sirajuddin SM, Stoneking M (2004). Independent origins of Indian caste and tribal paternal lineages. Curr Biol.

[B30] Wen B, Li H, Lu D, Song X, Zhang F, He Y, Li F, Gao Y, Mao X, Zhang L, Qian J, Tan J, Jin J, Huang W, Deka R, Su B, Chakraborty R, Jin L (2004). Genetic evidence supports demic diffusion of Han culture. Nature.

[B31] Ray BC (1989). Tribals of Orissa. The Changing Socio-economic Profile.

[B32] Trivedi R, Sitalaximi T, Banerjee J, Singh A, Sircar PK, Kashyap VK (2006). Molecular insights into the origins of the Shompen, a declining population of the Nicobar archipelago. J Hum Genet.

[B33] Shi H, Dong YL, Wen B, Xiao CJ, Underhill PA, Shen PD, Chakraborty R, Jin L, Su B (2005). Y- chromosome evidence of southern origin of the East Asian-specific haplogroup O3-M122. Am J Hum Genet.

[B34] Quintana-Murci L, Krausz C, Zerjal T, Sayar SH, Hammer MF, Mehdi SQ, Ayub Q, Qamar R, Mohyuddin A, Radhakrishna U, Jobling MA, Tyler-Smith C, McElreavey K (2001). Y-chromosome lineages trace diffusion of people and languages in southwestern Asia. Am J Hum Genet.

[B35] Lal BB (1956). Paleolithic from the Beas and Banganga vallelys Punjab. Ancient India.

[B36] Mohapatra GC (1975). Acheulian Element in Soan Culture Area. J Archaeol Soc Nippon.

[B37] Mohapatra GC, Deo SB, Paddaya K (1985). Cultural ecology of early man in India. Recent advances in Indian Archaeology.

[B38] Zide NH, Anderson GDS, Subbarao KV, Bhaskararao P (2001). The Proto-Munda Verb and Some Connections with Mon-Khmer. Yearbook of South Asian Languages and Linguistics.

[B39] Pinnow H-J, Shorto HL (1963). The position of the Munda languages within the Austroasiatic language family. Linguistic Comparison in Southeast Asia and the Pacific.

[B40] Underhill PA, Shen P, Lin AA, Jin L, Passarino G, Yang WH, Kauffman E, Bonne-Tamir B, Bertranpetit J, Francalacci P, Ibrahim M, Jenkins T, Kidd JR, Mehdi SQ, Seielstad MT, Wells RS, Piazza A, Davis RW, Feldman MW, Cavalli-Sforza LL, Oefner PJ (2000). Y chromosome sequence variation and the history of human populations. Nat Genet.

[B41] Cordaux R, Saha N, Bentley GR, Aunger R, Sirajuddin SM, Stoneking M (2003). Mitochondrial DNA analysis reveals diverse histories of tribal populations from India. Eur J Hum Genet.

[B42] Thangaraj K, Singh L, Reddy A, Rao V, Sehgal S, Underhill P, Pierson M, Frame I, Hagelberg E (2003). Genetic affinities of the andaman islanders, a vanishing human population. Curr Biol.

[B43] Ding YC, Wooding S, Harpending HC, Chi HC, Li HP, Fu YX, Pang JF, Yao YG, Yu JG, Moyzis R, Zhang Y (2000). Population structure and history in East Asia. Proc Natl Acad Sci USA.

[B44] Underhill PA, Passarino G, Lin AA, Shen P, Mirazon Lahr M, Foley RA, Oefner PJ, Cavalli-Sforza LL (2001). The phylogeography of Y chromosome binary haplotypes and the origins of modern human populations. Ann Hum Genet.

[B45] Fucharoen G, Fucharoen S, Horai S (2001). Mitochondrial DNA polymorphisms in Thailand. J Hum Genet.

[B46] Jobling MA, Tyler-Smith C (2003). The human Y chromosome: an evolutionary marker comes of age. Nat Rev Genet.

[B47] Wu XZ, Poirier FE (1995). Human evolution in China.

[B48] Jin L, Su B (2000). Natives or immigrants: modern human origin in East Asia. Nat Rev Genet.

[B49] Sambrook J, Fritsch EF, Maniatis T (1989). Molecular cloning: A laboratory manual.

[B50] YCC (The Y Chromosome Consortium) (2002). A nomenclature system for the tree of human Y-chromosomal binary haplogroups. Genome Res.

[B51] Butler JM, Schoske R, Vallone PM, Kline MC, Redd AJ, Hammer MF (2002). A novel multiplex for simultaneous amplification of 20 Y chromosome STR markers. Forensic Sci Int.

[B52] Excoffier L, Laval G, Schneider S (2005). An integrated software package for population genetics data analysis. Evolutionary Bioinformatics Online.

[B53] Bandelt H, Forster P, Rohl A (1999). Median joining networks for inferring intraspecific phylogenies. Mol Biol Evol.

[B54] Zhivotovsky LA, Underhill PA, Cinnioglu C, Kayser M, Morar B, Kivisild T, Scozzari R, Cruciani F, Destro-Bisol G, Spedini G, Chambers GK, Herrera RJ, Yong KK, Gresham D, Tournev I, Feldman MW, Kalaydjieva L (2004). The effective mutation rate at Y chromosome short tandem repeats, with application to human population-divergence time. Am J of Hum Genet.

