# Supplementary material for: Y-chromosome evidence suggests a common paternal heritage of Austro-Asiatic populations
Source: BMC Evol Biol. 2007 Mar 28;7:47. doi: 10.1186/1471-2148-7-47 (PMC1851701; doi:10.1186/1471-2148-7-47)
Supplement: Additional file 1 — Additional figures. It contains 3 figures; Fig S1) Map of India showing the area of sampling; S2) M-J Network of Y-STR haplotypes of O-M134* haplogroup; S3) Iso-frequency map of O-M69 in Asia. [file 1471-2148-7-47-S1.doc]

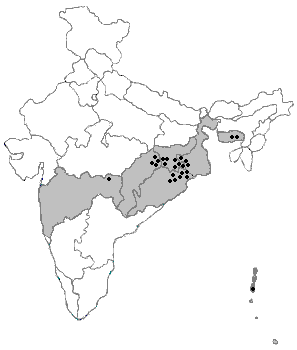


**Maharashtra**

**Orissa**

**Chattisgarh**

**Jharkhand**

**West Bengal**

**Meghalaya**

**Andaman and Nicobar**

**Nepal**

**China**

India

**Pakistan**

**Bhutan**

**Bangladesh**

**(Tibet)**

**Fig S1.** Map of India showing the areas of sampling. The black dots represent the regions of sampling within each state. The district-wise sampling details are furnished in Table 1.


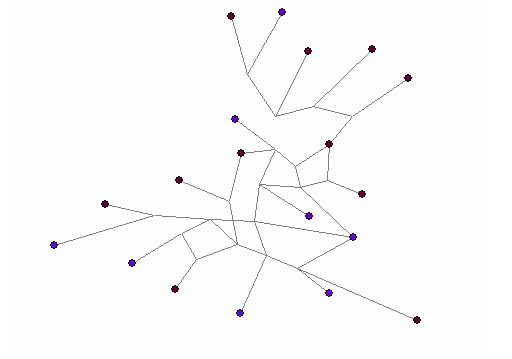


Khasi-Khmuic groups

Tibeto-Burman group

**Fig S2.** Median-Joining network of Y-STR haplotypes under the background of haplogroup O-M134*. Circles represent haplotypes with the area proportional to frequency. Microsatellite mutations are represented by black lines.


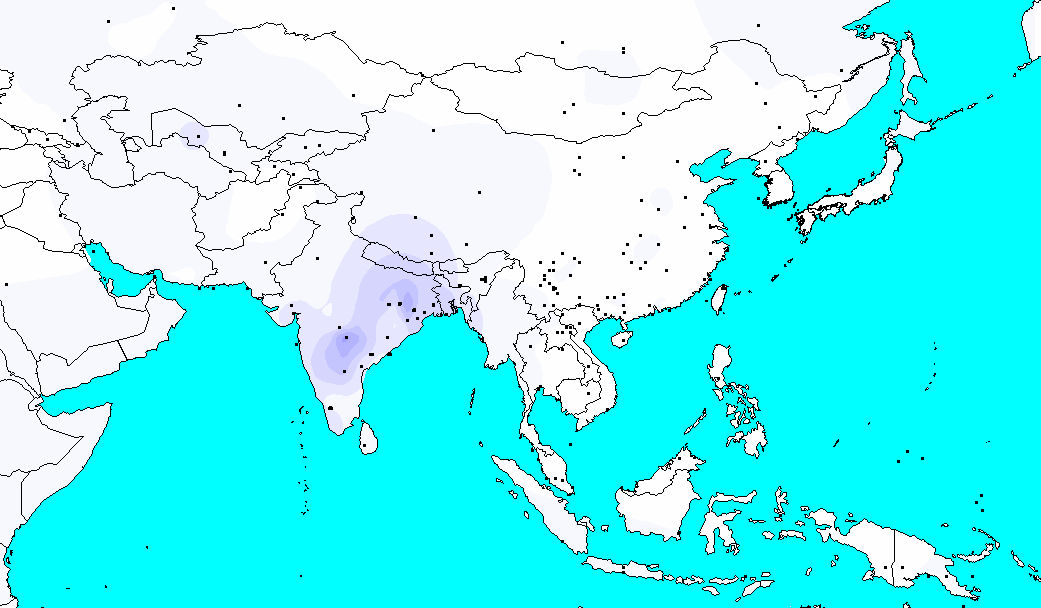

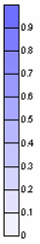


**Fig S3.** The isofrequency map portraying spatial distribution of haplogroup H-M69 in Asia and Oceana. The dots indicate the populations and the regions from where it was sampled. The sources for comparative data are cited in the legend of Figure 5.
